# White matter connectivity and social functioning in survivors of pediatric brain tumor

**DOI:** 10.1007/s11060-024-04724-0

**Published:** 2024-06-05

**Authors:** Matthew C. Hocking, Robert T. Schultz, Benjamin E. Yerys, Jane E. Minturn, Peter Fantozzi, John D. Herrington

**Affiliations:** 1 The Children’s Hospital of Philadelphia, Philadelphia, PA USA; 2 The University of Pennsylvania, Philadelphia, PA USA

**Keywords:** Pediatric brain tumor, Diffusion imaging, White matter, Social competence

## Abstract

**Objective:**

Survivors of pediatric brain tumors (SPBT) are at risk for social deficits, fewer friendships, and poor peer relations. SPBT also experience reduced brain connectivity via microstructural disruptions to white matter from neurological insults. Research with other populations implicates white matter connectivity as a key contributor to poor social functioning. This case-controlled diffusion-weighted imaging study evaluated structural connectivity in SPBT and typically developing controls (TDC) and associations between metrics of connectivity and social functioning.

**Methods:**

Diffusion weighted-imaging results from 19 SPBT and 19 TDC were analyzed using probabilistic white matter tractography. Survivors were at least 5 years post-diagnosis and 2 years off treatment. Graph theory statistics measured group differences across several connectivity metrics, including average strength, global efficiency, assortativity, clustering coefficient, modularity, and betweenness centrality. Analyses also evaluated the effects of neurological risk on connectivity among SPBT. Correlational analyses evaluated associations between connectivity and indices of social behavior.

**Results:**

SPBT demonstrated reduced global connectivity compared to TDC. Several medical factors (e.g., chemotherapy, recurrence, multimodal therapy) were related to decreased connectivity across metrics of integration (e.g., average strength, global efficiency) in SPBT. Connectivity metrics were related to peer relationship quality and social challenges in the SPBT group and to social challenges in the total sample.

**Conclusions:**

Microstructural white matter connectivity is diminished in SPBT and related to neurological risk and peer relationship quality. Additional neuroimaging research is needed to evaluate associations between brain connectivity metrics and social functioning in SPBT.

**Supplementary Information:**

The online version contains supplementary material available at 10.1007/s11060-024-04724-0.

Improved survival for pediatric brain tumors has heightened the urgency of understanding and addressing disease- and treatment-related sequalae. Survivors of pediatric brain tumor (SPBT) experience late effects across many domains [1, 2] that pose challenges as they navigate their social milieu [3]. SPBT experience social connectedness difficulties, such as fewer friends and social interactions compared to siblings and other childhood cancer survivors [4, 5].

While the causes of these social difficulties are poorly understood, they likely relate to tumor- and treatment-driven changes in brain structure and function. Biopsychosocial models that are grounded in social cognitive neuroscience [6, 7] emphasize the connectivity of brain networks that support essential social information processes [8]. Higher-order cognitive functions are implemented by brain structures connected via white matter tracts that develop through early adulthood [9, 10]. Structural connectivity is vital to network function and various social information processes, including face processing [11, 12] and empathy [13]. Notably, white matter integrity appears related to connectedness within real-world social networks [14].

Disruptions to network connectivity in childhood may lead to difficulties with social information processing [15] with effects on later social connectedness. Among adults, white matter lesions have been linked to decreased theory of mind abilities [16]. Reduced white matter integrity and altered white matter microstructure have been linked to social challenges in pediatric traumatic brain injury (TBI) [17] and autism spectrum disorder (ASD) [18–20].

SPBT are at risk for disrupted structural connectivity secondary to tumor-directed treatments and tumor-related sequelae [21]. Generally, higher radiation therapy (RT) dose and volume are related to greater white matter injury, with initial evidence that proton RT reduces white matter disruption compared to photon RT [22]. Neurosurgery and chemotherapy also confer risk for white matter abnormalities but to a lesser degree than RT [23]. These factors impair myelination and alter the balance between gray and white matter [10]. Furthermore, focal damage may affect global network dynamics. Early neuroimaging research showed reduced white matter volume in SPBT treated with craniospinal RT, with younger age and hydrocephalus as risk factors [24].

Emerging diffusion-weighted imaging (DWI) research has shown diminished white matter integrity in SPBT compared to controls [25–29]. Graph theory methods quantify the efficiency of processing information from distributed brain regions (e.g., global efficiency) and the level of local processing (e.g., modularity). During typical development, brain networks move from random network configurations to networks that optimize the balance between global and local processing. In a graph theory-driven DWI study, adult SPBT exhibited reduced global efficiency and average clustering coefficients between brain areas [30], suggesting significant structural network disruptions.

Given associations between brain connectivity and social cognition [31], microstructural injury to white matter networks may lead to deficits in basic social-affective processes [32] and social function among SPBT [33]. However, little research has addressed the link between white matter and social functioning in SPBT directly. This study’s objectives were to: 1) compare DWI connectivity metrics of SPBT and typically developing controls (TDC); 2) evaluate associations between neurological risk and connectivity metrics; and 3) determine associations between DWI metrics and indices of social functioning among SPBT. We hypothesized that a) SPBT would have decreased whole-brain connectivity compared to TDC; b) increased neurological risk would be associated with diminished connectivity; and c) reduced connectivity would be associated with more social challenges among SPBT.

## Methods

### Participants

Participants consisted of 38 English-speaking youth (ages 8–17 years): SPBT (N = 19) and TDC (N = 19). See Table 1 for sample descriptives. Participants were 13.7 years old, approximately 58% female, and nearly 30% were non-White. Groups were matched in terms of age (t(36) = 0.723, *p* = 0.474) and IQ, as indexed by the Differential Abilities Scale, Second Edition (DAS-II) General Conceptual Ability Score (t(35) = -1.344, *p* = 0.188). Participant IQ was in the Average range (*M* = 104; SD = 13.9). Groups did not differ in terms of sex, Race, or Ethnicity.

**Table 1:** Sample characteristics

Variables	Brain Tumor (n = 19) *n (%)* or M ± SD	Typically Developing (n = 19) *n (%)* or M ± SD	Test of Statistical Difference (*p*-value)
Age in years	14.05 ± 2.70	13.42 ± 2.63	*t* = 0.723 (*p* = .474)
Female sex	11 (57.9%)	11 (57.9%)	*Χ*^*2*^ = 0 (*p* = 1.0)
Race			*Χ*^*2*^ = 3.333 (*p* = .504)
Caucasian	12 (63.2%)	15 (78.9%)	
African-American	4 (21.1%)	2 (10.5%)	
Asian	1 (5.3%)	0	
Multi-ethnic	2 (10.5%)	1 (5.3%)	
Other	0	1 (5.3%)	
Hispanic/Latinx			*Χ*^*2*^ = 0.892 (*p* = 0.345)
Hispanic/Latinx	3 (15.8%)	1 (5.3%)	
Not Hispanic/Latinx	16 (84.2%)	16 (84.2%)	
Unreported	0	2 (10.5%)	
Income			*Χ*^*2*^ = 5.855 (*p* = .054)
< $34,000	5 (26.3%)	2 (10.5%)	
$34,000—$99,999	10 (52.6%)	5 (26.3%)	
> 99,999	3 (15.8%)	9 (47.4%)	
Highest Level of Maternal Education			*Χ*^*2*^ = 4.100 (*p* = .129)
High school or less	7 (36.8%)	3 (15.8%)	
Some college	5 (26.3%)	3 (15.8%)	
College degree or more	6 (31.6%)	12 (63.2%)	
Treatment factors			
Age at diagnosis	5.65 ± 3.15		
Time since diagnosis	8.45 ± 3.27		
Time since treatment completion	6.76 ± 3.88		
Tumor types			
Medulloblastoma	4 (21.1%)		
Ganglioglioma	4 (21.1%)		
Glioma	4 (21.1%)		
PNET	1 (5.3%)		
DNET	1 (5.3%)		
Pilocytic Astrocytoma	5 (26.3%)		
WHO grade			
I	13 (68.4%)		
II	1 (5.3%)		
III	0		
IV	5 (26.3%)		
Tumor Location			
Supratentorial	7 (36.8%)		
Infratentorial	12 (63.2%)		
Treatment			
Surgery only	9 (47.4%)		
Radiation only	0		
Chemo only	2 (10.5%)		
Surgery + radiation	1 (5.3%)		
Surgery + chemo	2 (10.5%)		
Radiation + chemo	0		
All three	5 (26.3%)		

*p*-value < 0.05* *p*-value < 0.01** *p*-value < 0.001***

SPBT included those with any combination of resection, chemotherapy and/or cranial RT, diagnosed at least 5 years prior, and completed all tumor-directed treatments at least 2 years prior. Exclusion criteria for SPBT included any genetic condition affecting neurocognitive functioning (e.g., Neurofibromatosis), developmental delay prior to brain tumor diagnosis, and visual defects uncorrectable through lenses (e.g., field cuts). Per caregiver report, one participant had an anxiety disorder diagnosis and one had an ADHD diagnosis. Survivors’ family history included autism and Asperger's (N = 3), ADHD (N = 1), and depression or anxiety (N = 2).

Participants were 5.6 years old at diagnosis and completed tumor-directed therapy 6.8 years prior. 63% (N = 12) had an infratentorial tumor and the sample reflected both low- and high-grade pathologies. The majority underwent surgical resection (89.5%; 13 gross total, 4 subtotal), 47.4% (N = 9) received chemotherapy, 36.8% (N = 7) had RT, and 47.4% (N = 9) had multimodal therapy (e.g., some combination of treatment modalities). Of those treated with cranial RT, 4 had proton and 3 had photon. Furthermore, 4 underwent focal RT while 3 received craniospinal RT. Two had post-operative hydrocephalus requiring shunt placement, two had hemiparesis, one had posterior fossa syndrome, and one had ataxia.

TDC were selected from a pool of 67 who completed the same research protocol at the Center for Autism Research (CAR) at the Children’s Hospital of Philadelphia (CHOP), using the same MRI scanner. These data were collected between June 2010 and October 2012 (SPBT data were collected between August 2016 and March 2018). Participants were selected on a case–control basis to match the SPBT on age, IQ, and sex. Exclusion criteria for TDC included a) visual defects uncorrectable with lenses; b) a history of TBI or other neurological abnormality; c) autism-like impairments on screening by study personnel; d) a first- or second-degree relative with ASD; and e) a DSM-IV-TR Axis I disorder or significant symptoms of ADHD or mood, anxiety, substance-related, or conduct disorders.

### Measures

#### Cognitive function

The DAS-II measured general cognitive ability [34]. The DAS-II provides norm-referenced overall cognitive ability scores (M = 100, SD = 15) that correlate highly with other IQ tests [34].

#### Social behavior

The Social Responsiveness Scale, Second Edition (SRS-2); [35] is a 65-item informant-report that evaluates the frequency of reciprocal social behaviors, communication, and repetitive and stereotypic behaviors. It yields a sex-normed total T-score with higher scores representing greater social challenges (M = 50, SD = 10). It has high internal consistency, test–retest reliability and inter-rater reliability, and strong associations with other measures of social difficulties [35].

The Vineland Adaptive Behavior Scale-Second Edition: Parent Rating Form (Vineland-II) [36] is a highly reliable measure of adaptive behavior. The Socialization domain standard score (M = 100, SD = 15) evaluated participant social functioning with higher scores reflecting better functioning.

The Children’s Communication Checklist-2 (CCC-2) [37] is a reliable parent-report measure of structural (grammar, syntax) and pragmatic (social reciprocity, gesture use) components of social communication. The Social Relations score (M = 10, SD = 3) measures pragmatic language with others and was used in analyses. Higher scores indicate better pragmatic language.

The Patient-Reported Outcomes Measurement Information System (PROMIS) Pediatric Peer Relationships-Short Form is a validated self-report measure of peer relationships [38]. Participants rate eight statements (i.e., I felt accepted by other kids my age) over the past 7 days on a 0–4 scale (0 = never, 4 = almost always). The measure yields a total T-score, where higher scores indicate better peer relationships. The self-report measure is correlated with peer-reported social acceptance [38]. Only SPBT participants completed this measure.

The Neurological Predictor Scale (NPS) [39] is generated through medical chart review and integrates treatment variables (e.g., cranial radiation) and history of neurological complications (e.g., hydrocephalus, seizure medication) into a single score on a scale of 0–11, with higher scores indicating greater neurological risk. The NPS has been related to neurocognitive functioning [40] and DWI metrics [30].

### Procedures

Study procedures were approved by the CHOP institutional review board. Potentially eligible SPBT were identified through electronic medical records and sent a letter describing the study. Families were contacted by phone to conduct a verbal screening to determine eligibility. Those meeting eligibility criteria and interested in participating were invited to a one-time, in-person evaluation. SPBT participants were given the option of completing a 1-h research MRI scan. Written informed consent was obtained from parents and child assent was obtained. The protocol with SPBT mirrored that used with TDC as part of separate studies measuring social function with a core set of measures. Parents completed informant reports while youth completed the assessment. SPBT and TDC data were combined for analyses. 207 SPBT were contacted and 97 were screened. Of those screened, 90 met criteria, 54 completed the cognitive assessment, and 23 completed neuroimaging. 4 SPBT had excessive movement during the scan, leaving a total of 19. There were no differences on main study measures (DAS-II GCA, SRS-2, CCC-2, Vineland Socialization) between SPBT who completed an MRI scan and those who did not.

#### MRI scanning

All scans were conducted on a Siemens Verio 3-Tesla. Diffusion-weighted imaging data consisted of a 30-direction sequence (80 axial slices, 2 mm isotropic voxels, b^0^ = 1000, TR/TE/Flip Angle = 11 s/76.4 ms/180 degrees). High-resolution structural data also were collected on all participants (TR/TE/Flip Angle/Voxel Size parameters of 1900 ms/ 2.54 ms/90 degrees/0.8x.8. × 9 mm), and used to register diffusion data into atlas space (see below).

### Data processing

**Fig. 1:**
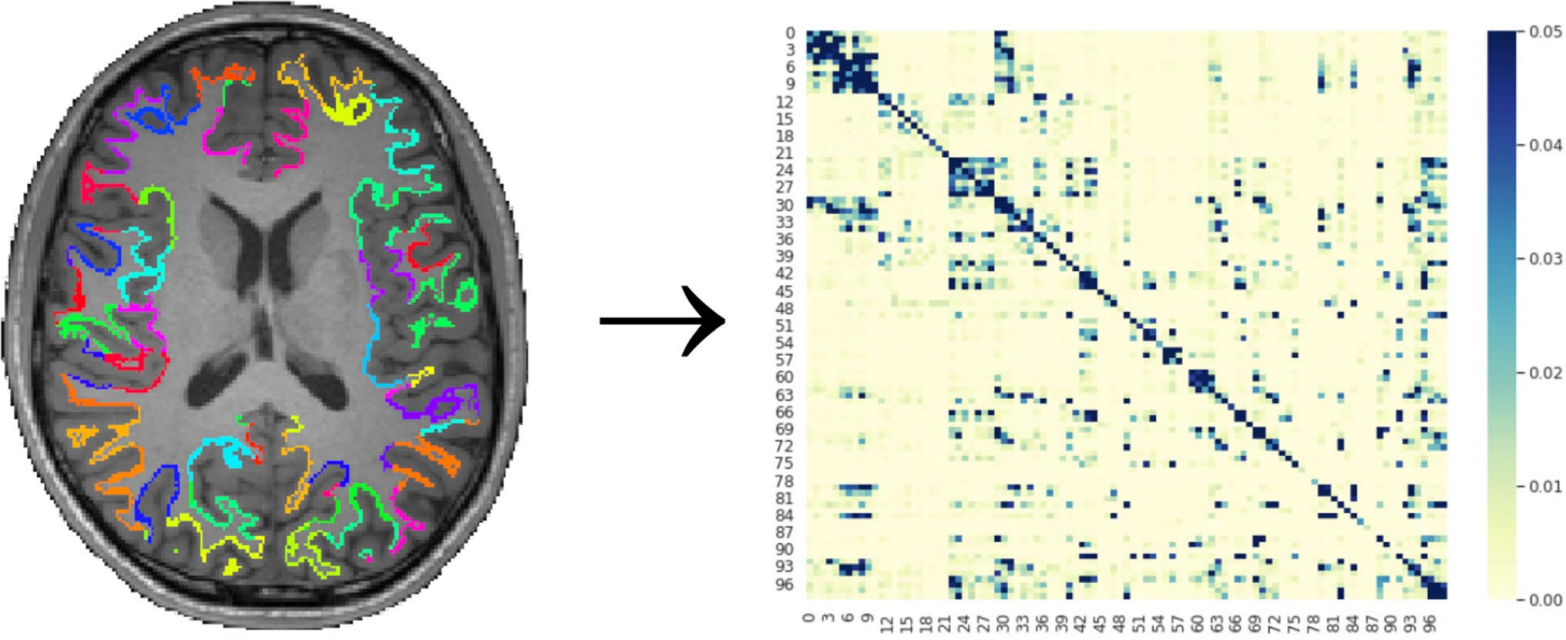
**Connectivity Matrix Generation [**Note. Illustration of connectivity matrix generation for one participant. The Schaefer et al. template was registered to each participant structural MRI. Using white matter segmentations and commands from Freesurfer, the portion of each parcellation overlapping with the white matter boundary was identified. These parcellation boundaries were then used as seed areas for probabilistic tractography using F ProbtrackX. An adjacency matrix was then generated for all possible pairs of areas. Graph theory metrics were then calculated for each participant’s adjacency matrix, which were used for group-level statistics.]

All data were corrected for motion and eddy currents using the program eddy_correct from the fMRIB Software Library (FSL) [41]. Connectivity data were estimated via probabilistic tractography, using the FSL programs bedpostx and probtrackx [42]. Probtrackx uses tensor information to develop probabilistic models of fiber pathways between seed and target areas. Bedpostx was configured up to two crossing fibers per voxel via a stick function deconvolution model. Our approach to identifying seed regions for probabilistic tractography follows established approaches [43]. Each participant's high-resolution structural MRI volume was segmented into gray and white matter using Freesurfer [44]. Using Freesurfer, we isolated portions of the Schaefer et al. parcellation [45] that overlap with their corresponding white matter boundaries (see Fig. 1). These boundaries were then transformed to each participant’s diffusion space using FSL’s program FLIRT [41]. All possible parcel pairings were included as seed and target areas, resulting in connection probability matrices for all parcels, for everyone in the sample. Because the Schaefer et al. template focuses on gray matter, the portions of each parcel on the white matter boundary (derived from per-participant Freesurfer segmentations [46]) were used as seed regions.

## Statistical analyses

We evaluated hypotheses via graph theory analyses [47]. Graph theory provides several scalar metrics reflecting integration and segregation between networks and nodes (i.e., brain areas). Analyses focused on average connectivity strength (reflecting overall connectivity), global efficiency (a measure of network integration), assortativity (measure of the extent to which nodes in a sub-network associate with other nodes in the sub-network, and the resilience of the network against damage to main components), average clustering coefficient (measure of segregation, where high values indicate high levels of within-network, rather than global, connectivity), modularity (the degree to which nodes segregate into subnetworks), and betweenness centrality (the number of shortest paths between pairs of brain areas that also pass through any given area, averaged across nodes in a network). We predicted that these values would be decreased in the SPBT group, except for modularity and betweenness centrality (which typically increase when global connectivity decreases). All graph theory statistics were estimated using the Brain Connectivity Toolbox [47]. Binary input versions of graph theory formulas were used when estimating degrees and betweenness centrality; the rest used continuous input versions. T-tests compared imaging metrics between SPBT and TDC. The False Discovery Rate (FDR) correction controlled for family-wise error in analyses [48]. Effect sizes were estimated using Cohen's d, with scores of 0.2, 0.5, and 0.8 reflecting small, medium and large effects, respectively [49].

Evaluating brain connectivity among SPBT poses a dilemma – while global connectivity disruptions are of intrinsic importance, they would be expected based on the presence of tumors, resection, and associated neurosurgical procedures. We addressed this issue via a sensitivity analysis where we removed those SPBT with obvious morphological differences (i.e., readily identifiable from structural scans; n = 6), and re-running group comparisons. Pearson correlations evaluated associations between medical factors and imaging metrics within SPBT and associations between imaging metrics and indices of social function.

## Results

### Preliminary analyses

SPBT demonstrated higher levels of social challenges than TDC across all measures of social functioning, including the SRS-2 Total Score, t(35) = 4.366, *p* =  < 0.001, the Vineland-2 Socialization Score, t(33) = -2.410, *p* = 0.029, and the CCC-2 Social Relations score t(32) = -3.862, *p* =  < 0.001, with effect sizes ranging from medium to large. However, scores across measures for both groups were in the average range. See Table 2.

**Table 2:** Differences in social functioning between SPBT and TDC

Domain	SPBT Mean (SD)	SPBT Range	TDC Mean (SD)	TDC Range	T Value (1, 36)	Cohen’s d (CI)
DAS-II IQ Score	101.28 (12.51)	74 — 124	107.37 (14.88)	82 — 136	- 1.344	- 0.442 (- 1.092, 0.214)
PROMIS Peer Relationships Score	45.42 (7.79)	29.24 — 56.82	-	-	-	-
SRS-2 Total T Score	52.17 (8.58)	42 — 76	42.53 (4.26)	38 — 53	4.293***	1.436 (0.701, 2.155)
Vineland Socialization Score	101.18 (22.59)	62 — 140	114.72 (7.47)	101 — 129	- 2.354*	- 0.815 (- 1.501,—0.118)
CCC-2 Social Relations Score	9.28 (2.89)	4 — 13	12.13 (0.62)	11 — 13	- 4.081***	- 1.327 (- 2.066,—0.571)

*p*-value < 0.05* *p*-value < 0.01** *p*-value < 0.001***

Within the full sample, average connectivity strength was associated with IQ (r = 0.366; *p* = 0.026). Among SPBT, average connectivity strength (r = 0.562; *p* = 0.015), global efficiency (r = 0.614; *p* = 0.007), and modularity (r = 0.692; *p* = 0.001) were significantly associated with IQ. Age at evaluation was positively associated with global efficiency (r = 0.458; *p* = 0.049) among SPBT (See Table 3).

**Table 3:** Correlations between white matter connectivity and social functioning among SPBT

Imaging Metrics	Age at Evaluation	NPS Scores	DAS-II IQ	PROMIS Peer Relationships Scale Self-Report	SRS-2 Total Score	Vineland-II Socialization Score	CCC2 Social Relationships Score
Average Connectivity Strength	.386	-.541*	.562*	-.158	-.022	-.259	-.324
Global Efficiency	.458*	-.568*	.614**	-.147	-.165	-.187	-.194
Assortativity	.352	.012	-.039	.477*	-.012	.269	-.209
Clustering Coefficient	.010	.133	-.017	-.136	.518*	-.151	-.407
Modularity	.377	-.028	.692**	.015	-.409	.272	-.212
Betweenness Centrality	.258	-.331	.101	-.166	-.153	.228	.062

*p*-value < 0.05* *p*-value < 0.01** *p*-value < 0.001***

### Diffusion weighted imaging by group

Table 4 presents imaging metrics by group. Compared to TDC, SPBT demonstrated decreased average connectivity strength, global efficiency, assortativity, and clustering coefficient (*p*s < 0.01) with large effect sizes. Modularity (*p* = 0.038) and betweenness centrality (*p* < 0.001) were significantly increased in SPBT with medium to large effect sizes.

**Table 4:** Differences in white matter connectivity between SPBT and TDC

Imaging Metrics	SPBT Mean (SD)	TDC Mean (SD)	T Value (1, 36)	Cohen’s d (CI)
Average Connectivity Strength	0.257 (0.036)	0.324 (0.039)	-5.518***	- 1.790 (-2.539, -1.024)
Global Efficiency	0.005 (> 0.001)	0.006 (> 0.001)	-3.430**	- 1.113 (-1.792, -0.421)
Assortativity	-0.050 (0.011)	-0.039 (0.008)	-3.562***	- 1.156 (-1.838, -0.459)
Clustering Coefficient	0.0006 (< 0.001)	0.0007 (< 0.001)	-5.385***	- 1.908 (-2.741, -1.052)
Modularity	0.439 (0.023)	0.424 (0.020)	2.150*	0.698 (0.037, 1.349)
Betweenness Centrality	13.815 (4.278)	8.143 (2.265)	5.108***	1.657 (0.907, 2.390)

*p*-value < 0.05* *p*-value < 0.01** *p*-value < 0.001***

### Medical characteristics and imaging metrics

Average connectivity strength was related to recurrence (t = -3.276, *p* = 0.004), multimodal treatment (t = -2.958, *p* = 0.009), and chemotherapy (t = -2.741, *p* = 0.014). Global efficiency was related to recurrence (t = -2.599, *p* = 0.019) and multimodal treatment (t = -2.847, *p* = 0.011), but not chemotherapy (t = -2.112, *p* = 0.05). Higher NPS scores were related to reduced average strength and global efficiency. Each of these effects remained significant when applying family-wise error correction via FDR and when removing SPBT with gross morphological differences from resection. See Table 3. Age at diagnosis, time since diagnosis, and time since treatment completion were unrelated to imaging metrics.

### DWI metrics and social functioning

Among the entire sample, increased social challenges on the SRS-2 were associated with reductions in average connectivity strength (r = -0.420; *p* = 0.010) and global efficiency (r = -0.379; *p* = 0.021). Additionally, higher levels of modularity were associated with worse social communication on the CCC2 (r = -0.365; p = 0.034) and higher levels of assortativity were associated with better social relations on the Vineland-II (r = 0.445, *p* = 0.007) (see Supplemental Table 2).

Among SPBT, increased assortativity was associated with better peer relationship scores on the PROMIS Pediatric Peer Relationships Scale (*r* = 0.477, *p* = 0.045). Furthermore, higher clustering coefficient was associated with greater social challenges on the SRS-2 (*r* = 0.518, *p* = 0.040). Among TDC, higher levels of assortativity were related to better Socialization scores on the Vineland (*r* = 0.475, *p* = 0.046). There were no other significant associations between imaging metrics and other primary outcomes among TDCs.

## Discussion

Prior research has identified both disrupted white matter connectivity [21, 24–29] and social impairments [3, 4] in SPBT, yet few studies have attempted to connect these issues. Findings from this initial study of white matter connectivity and social functioning in SPBT offer preliminary support for this framework. Results suggest decreased global connectivity, as evidenced by global efficiency and average strength, reduced network resilience, as measured by assortativity, and increased within-network connectivity, as evidenced by increased modularity and betweenness centrality, in SPBT compared to age-, sex-, and IQ-matched TDC. Higher levels of neurological risk were associated with decreased connectivity. Further, higher assortativity was associated with better peer relationships among SPBT, suggesting that those with more resilient white matter networks characterized by stronger connections among nodes [47] experience greater social acceptance. Additionally, among SPBT higher within-network, compared to global, connectivity (clustering coefficient) was associated with more social challenges.

The pattern of connectivity seen in this study with school-age SPBT implies diminished global network organization and is consistent with a prior study employing graph theory approaches in adult SPBT [30]. Collectively, these findings suggest decreased processing efficiency across the brain and increased reliance on sub-network processing in SPBT. Further, connectivity metrics in both studies appear associated with neurological insults. Typical neurodevelopment involves progressing to networks that balance between integration and segregation between networks of brain areas [30], and disruptions in development from tumor and treatments likely contribute to the differences in SPBT connectivity. Longitudinal research is needed to document these neurodevelopmental processes in SPBT.

Study findings contribute to a growing body of research concerned with neurophysiological explanations underlying social behavior and social information processing among SPBT [50]. Greater connectivity between sub-network nodes, as measured by assortativity, was related to better self-reported peer relationships. However, other connectivity metrics were unrelated to measures of social behavior or relationships. These findings contrast somewhat with prior research linking white matter connectivity and social function in both typical and clinical populations [17–20]. Consistent with hypothesized models [33], network connectivity and reduced network segregation likely underlie the development of domains of social cognition, which in turn promote increased connections with others [31].

Methodological strengths of this study include using a case-controlled design that matched SPBT to TDC in terms of age, IQ, and sex, and employing graph theory analyses for DWI data, which is relatively novel in research on SPBT. Limitations include a relatively small sample with complete imaging and social behavior data and a heterogenous sample of SPBT mixed in tumor type, location, and treatment combination. These issues reduced power to evaluate each of these factors independently. The TDC group also was relatively high functioning, which may have contributed to observed group differences.

This study offers an initial link between the neurophysiological changes seen in white matter in SPBT and their social difficulties. Additional studies with larger samples and longitudinal designs are needed to determine within-person associations between developmental (e.g., age at diagnosis) and medical (e.g., tumor location, radiation factors) variables, white matter network characteristics, and social information processing and social behavior in SPBT over time. Such research may identify the mechanistic factors that affect the variability in survivor social functioning and inform remediation interventions.

### Supplementary Information

Below is the link to the electronic supplementary material.

Supplementary file1 (DOCX 203 KB)

## Data Availability

The data that support the findings of this study are available upon request from the corresponding author. The data are not publicly available due to their containing information that could compromise the privacy of the research participants.
