# Supplementary material for: White matter connectivity and social functioning in survivors of pediatric brain tumor
Source: J Neurooncol. 2024 Jun 5;169(2):247–56. doi: 10.1007/s11060-024-04724-0 (PMC11341593; doi:10.1007/s11060-024-04724-0)

**Supplemental Tables:**

**Supplemental Table 1: Correlations between white matter connectivity and social functioning among full sample**

| **Imaging Metrics** | **Age at Evaluation** | **DAS-II IQ** | **SRS-2 Total Score** | **Vineland-II Socialization Score** | **CCC2 Social Relationships Score** |
| --- | --- | --- | --- | --- | --- |
| Average Connectivity Strength | .027 | **.366*** | **-.420**** | .154 | .293 |
| Global Efficiency | .106 | .306 | **-.379*** | .061 | .212 |
| Assortativity | .296 | .247 | -.324 | **.445**** | .171 |
| Clustering Coefficient | -.132 | .213 | -.169 | **.367*** | .257 |
| Modularity | .275 | .086 | -.015 | .060 | **-.365*** |
| Betweenness Centrality | .162 | -.079 | .289 | -.169 | -.327 |

p-value < 0.05* p-value <0.01** p-value < 0.001***

**Supplemental Table 2: Correlations between white matter connectivity and social functioning among TDC**

| **Imaging Metrics** | **Age at Evaluation** | **DAS-II IQ** | **SRS-2 Total Score** | **Vineland-II Socialization Score** | **CCC2 Social Relationships Score** |
| --- | --- | --- | --- | --- | --- |
| Average Connectivity Strength | -.082 | .129 | -.133 | -.001 | .137 |
| Global Efficiency | -.095 | -.056 | -.109 | -.149 | .180 |
| Assortativity | **.523*** | .385 | -.031 | **.475*** | -.108 |
| Clustering Coefficient | -.094 | .284 | .264 | .431 | .120 |
| Modularity | .103 | -.291 | .035 | .070 | -.062 |
| Betweenness Centrality | -.159 | .087 | -.194 | -.303 | -.114 |

p-value < 0.05* p-value <0.01** p-value < 0.001***

**Supplemental Table 3: Differences in white matter connectivity between SPBT treated with and without radiation**

| **Imaging Metrics** | **Radiation**  **(SD)** | **No Radiation (SD)** | **T Value**  **(1, 17)** | **Cohen’s d**  **(CI)** |
| --- | --- | --- | --- | --- |
| Average Connectivity Strength | 0.247 (0.029) | - 1. (0.040) | - 0.907 | - 0.431  (- 1.368, 0.518) |
| Global Efficiency | 0.0046 (>0.001) | 0.0050 (>0.001) | - 0.993 | - 0.472  (- 1.411, 0.480) |
| Assortativity | - 0.050 (0.012) | - 0.049 (0.011) | - 0.128 | - 0.061  (- 0.992, 0.873) |
| Clustering Coefficient | 0.00057 (<0.001) | 0.00055 (<0.001) | 0.612 | 0.311  (- 0.695, 1.306) |
| Modularity | 0.442 (0.018) | 0.438 (0.026) | 0.344 | 0.164  (- 0.772, 1.095) |
| Betweenness Centrality | 13.626 (4.187) | 13.926 (4.511) | - 0.143 | - 0.068  (-1.000, 0.865) |

p-value < 0.05* p-value <0.01** p-value < 0.001***

**Supplemental Figures:**

**Supplemental Figure 1: PROMIS peer relationships score by assortativity among SPBT**


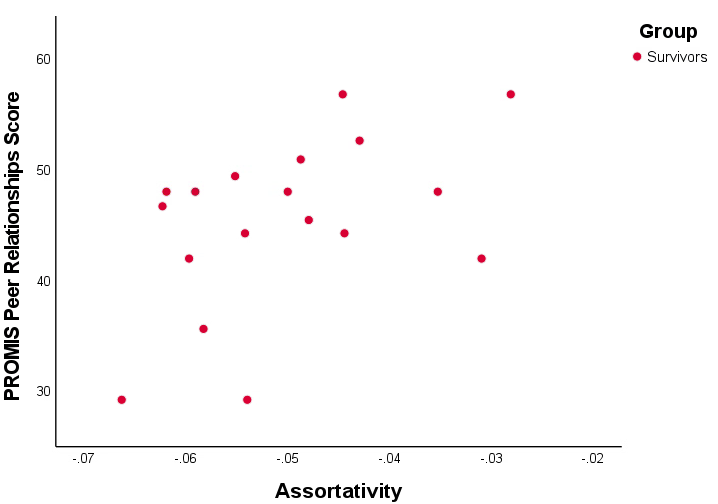


**Supplemental Figure 2: SRS-2 total score by clustering coefficient among full sample**


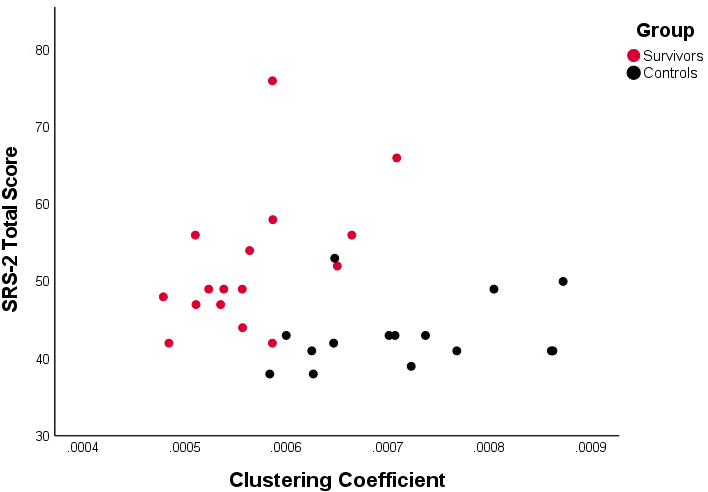


**Supplemental Figure 3: SRS-2 total score by assortativity among full sample**


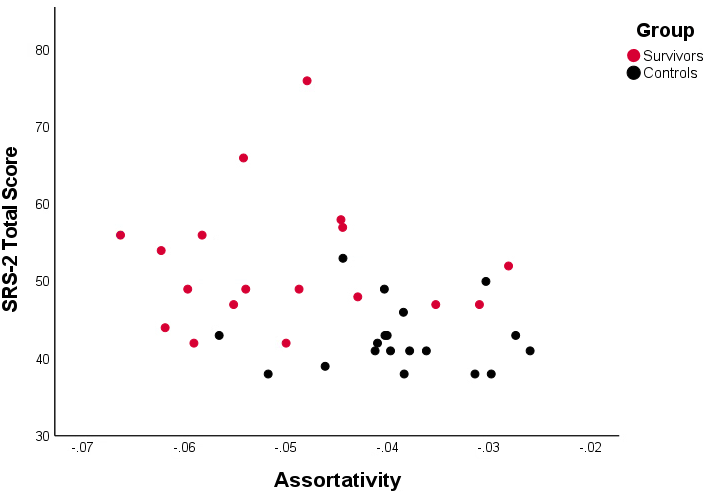


**Supplemental Figure 4: Vineland-II socialization score by assortativity among full sample**

**
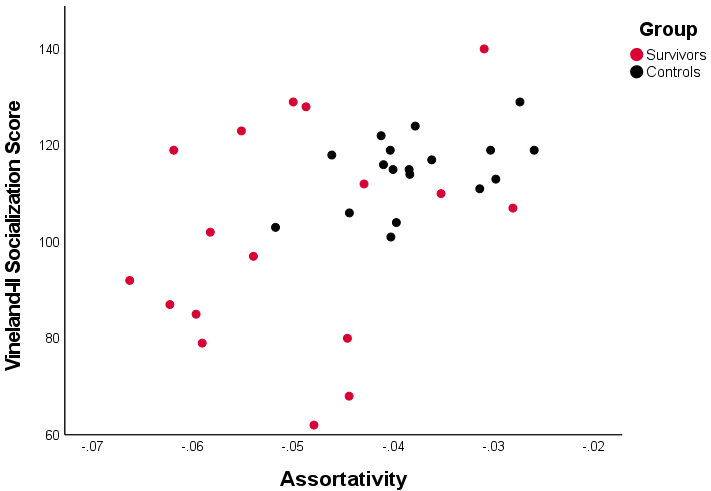
**

**Supplemental Figure 5: SRS-2 total score by global efficiency among full sample**

**
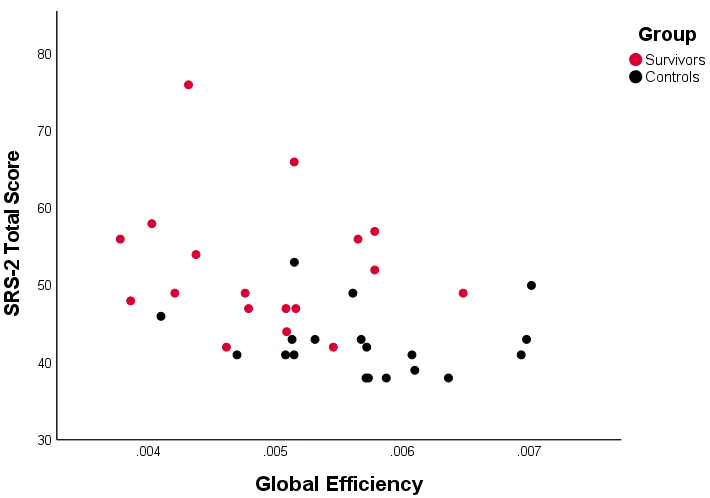
**

**Supplemental Figure 6: SRS-2 total score by average connectivity strength among full sample**

**
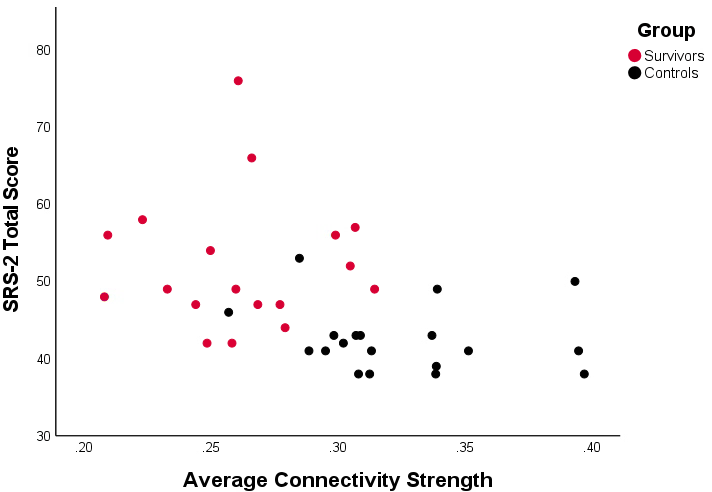
**

**Supplemental Figure 7: Vineland-II socialization score by clustering coefficient among full sample**


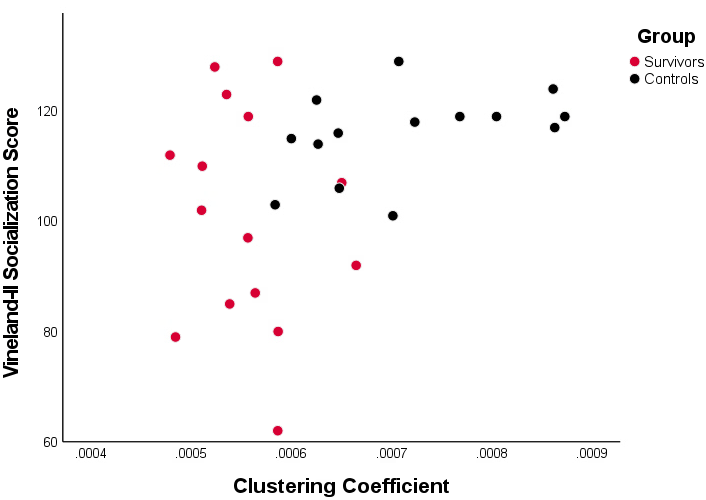

Supplement: Supplementary file 1 — Supplementary file1 (DOCX 203 KB) [file 11060_2024_4724_MOESM1_ESM.docx]
